# Radiotherapy teaching during COVID-19: An emergency teaching response

**DOI:** 10.4102/sajo.v6i0.251

**Published:** 2022-12-08

**Authors:** Hester Burger, Nanette Joubert, Bridget Wyrley-Birch, Natalia Vowles, Antonella Fogliata, Theresa Binz, Jeannette D. Parkes

**Affiliations:** 1 Department of Radiation Medicine, Faculty of Health Sciences, University of Cape Town, Cape Town, South Africa; 2 Medical Affairs Division, Varian Medical Systems, Cape Town, South Africa; 3 Department of Medical Imaging and Therapeutic Sciences, Faculty of Health and Wellness Sciences, Cape Peninsula University of Technology, Cape Town, South Africa; 4 RadiQa Developments, Bellinzona, Switzerland; 5 Humanitas Research Hospital, Milan-Rozzana, Italy; 6 Planning for Africa, Abu Dhabi, United Arab Emirates

**Keywords:** COVID-19, remote teaching, radiotherapy training, hybrid training, e-learning, virtual reality, treatment planning, LMIC, Africa

## Abstract

**Background:**

The coronavirus disease 2019 (COVID-19) pandemic resulted in more than six million deaths in the first two years, a third of the estimated number of cancer-related deaths during this time. It directly impacted radiotherapy training in Africa.

**Aim:**

This study evaluated the changes applied to the Access to Care Cape Town Radiotherapy training programme during the pandemic.

**Setting:**

The training platform prior to March 2020 was used as a baseline and compared with the programme status in January 2022, representing the emergency teaching model.

**Methods:**

Five themes were investigated: computer hardware and software changes; e-Learning resources; programme and curriculum changes; challenges experienced and alignment with modern medical education principles.

**Results:**

Reconfiguration of the computer laboratories was required, including additional computer monitors, web cameras and headsets, as well as installation of screen recording and teleconferencing software. The Eclipse^TM^ radiotherapy treatment planning laboratory was reconfigured for remote student access, with simultaneous monitoring by local assistants. Online learning was augmented by adding the University of Cape Town Vula^TM^ system as resource, and courses restructured for delivery of short blocks. Five new courses were developed, including collaborations with international training partners, showing good alignment with the principles of modern medical education.

**Conclusion:**

Reconfiguration was performed at a manageable cost but required a high level of information technology support. Connectivity and bandwidth issues remain a challenge, as well as online engagement.

**Contribution:**

Despite these challenges, the virtualisation allowed for continued training between March 2020 and December 2021, with 18 departments attending remote teaching courses.

## Introduction

It has been more than two years since the start of the coronavirus disease 2019 (COVID-19) pandemic, and while staggering in terms of its global impact on both economy and human life, the number of deaths reported is still only a third of the estimated number of cancer-related deaths (19.8 million) in the same time period.^1,2^ Cancer mortality rates may be even higher than these estimates because of the catastrophic impact of the pandemic. The World Health Organization (WHO) reports that cancer screening, diagnosis and treatment have suffered in an unprecedented way because of health systems’ requirement to respond to the influx of COVID-19 patients. The WHO Global Pulse Survey showed that, in the last quarter of 2021, there was disruption of 5% to 50% in screening and treatment of cancer in European countries. Although the situation has improved since then, 44% of countries have reported an increase in service backlogs with a long-term knock-on effect being predicted.^3^ Similar reports from Africa indicate that more than 75% of cancer care service providers report decreases in services offered between June and August 2020 and although relatively few COVID-19-related deaths were reported among cancer patients, severe disruptions to services occurred.^4^

Africa has always faced challenges in terms of cancer care, with lower numbers of oncology centres and treatment machines per person compared with other continents. There is no region in Africa with more than 3 megavoltage radiotherapy (RT) machines per million population and several countries have no access to RT at all. This is compared with North America with 11 machines/million population and Western Europe with a reported 7 machines/million population.^5^ Radiotherapy forms an essential part of cancer care, with approximately half of all cancer patients requiring RT at some point during their treatment and this number may be even greater in low- and middle-income countries (LMICs) where more patients present with advanced disease.^6^ Several international programmes have been launched over the last decade to address the need for additional RT machines in Africa, resulting in a steady increase in the number of megavoltage units being installed on the continent^7^ and this in turn, has resulted in increased pressure to teach and train RT professionals to safely plan and operate the modern units and create safe clinical treatment plans.^8^

The Access to Care (A2C) Cape Town radiotherapy training programme was launched in 2015 to address this training need on the continent. The focus of the programme was to allow teams of radiation therapy professionals (radiation oncologist [RO], medical physicist [MP] and radiation therapist [RTT] from the same department) to attend practical training in Cape Town over a 17-day period, to facilitate the transition from basic RT techniques utilising 2D planning and cobalt machines, to more modern techniques utilising CT-based 3D conformal radiotherapy (3D CRT).^9^ The increased conformity that is achievable with 3D CRT results in a more homogeneous dose delivery to the target volumes as well as a reduction in dose to critical organs at risk. The potential to improve local control as well as toxicity is directly beneficial to patients and is well described in the International Atomic Energy Agency’s (IAEA) Technical Document 1588.^10^ The IAEA TECDOC 1588 also highlights the need for safe implementation of the techniques to reduce risk of errors, making training essential.

Prior to the pandemic, A2C training was based on a hybrid e-learning and in-classroom teaching model. Pre-course learning and concept revision was performed through the LäraNära online training platform (https://online.laranara.se/), made available through Varian Education.^11^ LäraNära is a Swedish-based RT distance learning platform that allowed participants access to online modules to review the principles of RT equipment, radiation beam characteristics, imaging, contouring, treatment planning, delivery techniques and radiation protection prior to the course. Participants were required to complete a test at the end of each module. The pre-course learning was followed by 17 days of in-person classroom-based training, with access to a virtual reality radiotherapy simulation system (VERT^TM^; Vertual Ltd, United Kingdom [UK], https://www.vertual.co.uk/), a radiotherapy treatment planning (RTP) laboratory (Eclipse^TM^, Varian Medical Systems, United States [US]), and demonstrations in the clinical department. The curriculum followed a modified version of the RT treatment chain with reference to specific clinical sites, including prostate, cervix and breast.^12^ Teams were given the opportunity to attend didactic lectures, contouring and planning demonstrations and to attend up to 45 h of supervised practical planning and contouring, while developing clinical protocols to safely implement the 3D CRT techniques in their home departments. Details of the programme can be found in the previous article published in 2021.^9^

The identification of the first COVID-19 patient in South Africa on 05 March 2020, and the subsequent declaration of a National State of Disaster on 15 March 2020, resulted in a complete travel ban to and from South Africa.^13^ The team from Uganda that was in training in South Africa at the time had to be repatriated immediately and could not complete the last week of training.

With no in-person training possible for the foreseeable future, the decision was taken to immediately modify the platform to allow for emergency remote teaching to complete the training for the Ugandan team and allow future courses to take place. The restructuring process also offered the opportunity to reassess and modernise the programme itself in terms of the principles of modern medical education as described by Filetti et al.^14^:

- *Interactivity* – activities should promote interaction with students, increase curiosity and boost engagement.
- *Bidirectionality* – application of knowledge to challenging problems, with key focus on collaboration and continuous feedback between educators and students, as well as peer-to-peer.
- *Blendedness* – utilisation of a mix of new technologies and traditional methods to keep participants engaged.
- *Transnationality* – encourage understanding of cultural diversity through international cooperation.
- *Up-to-datedness* – discourage material recycling from year to year, material should be refreshed continuously to ensure relevance and current practice planning strategies.

Studies have shown that student comprehension and engagement increases when students are exposed to live-stream lectures, compared with more static pre-recorded lectures of the same format, identifying entertainment as the main factor conducive to academic achievement for the current generation of students. Special attention was therefore given to increasing the entertainment factor during training through live-streaming and creation of more entertaining video training material.^15^ Training would still be carried out in the team context, to support the critical need for multidisciplinary team learning.^16^

This study aims to retrospectively analyse and document all changes made to the Access to Care Cape Town training programme between March 2020 and January 2022, to deal with the teaching challenges resulting from the COVID-19 pandemic.

## Study methodology

The status of the programme as previously described (2015–2019) was used as baseline, representing the in-person training model.^9^ An investigation was performed to determine the status in January 2022, considered to be representative of the emergency remote teaching model. This included an assessment of the computer equipment in both classrooms, comparison of the programmes of all courses offered between March 2020 and January 2022, and reviews of the training material changes and feedback from participants and faculty regarding challenges experienced. The investigation focused on five themes:

- computer hardware and software changes required to facilitate remote teaching, including cost implications
- E-learning resource utilisation
- programme and curriculum changes applied
- key challenges experienced by faculty and participants
- alignment with the principles of modern medical education as described by Filletti et al.^14^

### Ethical considerations

The study was performed with approval from the University of Cape Town Human Research Ethics Committee (HREC ref. 829/2019). Informed consent was obtained from all involved teaching faculty and participants to use feedback and comments anonymously for research purposes.

## Results

### Computer hardware and software changes

#### Teleconferencing facilities

Several commercially available teleconferencing solutions were investigated for live streaming of lectures and general communication with participants. The Zoom^TM^ platform was selected as it allowed easy access for participants, without the need to register a Zoom account (https://zoom.us/). Bandwidth and speed testing also showed that it functioned well with limited bandwidth availability, with the option to stop individual video feeds as needed. Session facilitators set up meetings through registered Zoom accounts to allow for up to 7-h long meetings each day. A key feature of the platform is the ability to break into smaller groups for some sessions, facilitated through the Breakout Rooms option, required for treatment planning teaching.

Both classrooms were upgraded to have two screens (monitors) available per computer. This made slide sharing and simultaneous monitoring of Zoom easier, as participants could be viewed on one monitor, while material was being displayed and streamed to participants on the second monitor. This limits interruptions because of application switching during teaching. Web cameras were installed to facilitate good sound and video transmission, within bandwidth limitations, and headsets made available.

#### Recording facilities

Where lecturers were not available on the specific day of teaching or wanted material to be available at a later stage, video recordings were made using the free Open Broadcaster Software (OBS, https://obsproject.com/). These recordings could then be streamed live through the Zoom platform, inserted as clips into PowerPoint slides, or uploaded to Vula™ for viewing at a later stage.

#### The VERT^TM^ radiotherapy simulation system

The VERT^TM^ radiotherapy simulation system is a commercial computer system that allows for 2D or 3D visualisation of RT and medical physics quality assurance equipment and processes. It simulates the functions of the equipment in question and allows for demonstration of normal and fault scenarios without risk to clinical equipment.^9^ The system was installed in 2015 and runs on a closed network because it is still utilising Windows 7 as operating system, which is considered a network security risk. Live streaming from the platform was therefore not possible. As an interim measure, screenshots and video recordings were made of several scenarios on VERT^TM^ and embedded into PowerPoint slides to be used during training. The OBS software was installed to facilitate recordings.

#### Strategic thinking whiteboard exercises

A physical white board team exercise was previously used to demonstrate processes involved in procurement including department design, staffing, networking, radiation shielding, quality assurance and budget management. Each team was given a different scenario and given the challenge of designing a department and presenting their designs to teaching faculty and other teams. A computer-based solution for this exercise had to be found. This was carried out using pre-populated PowerPoint and Excel spreadsheet templates.

#### Radiotherapy treatment planning laboratory

The RTP laboratory used a cloud-based interface between local Dell Wyse Thin Client^TM^ workstations and the main server in Switzerland, set up and maintained by Varian. Communication between the local clients and the main server was achieved using the West Africa Cable System (WACS) through an agreement with the South African National Research and education Network (SANReN), as part of the University of Cape Town’s quota.

The server hosts multiple virtual instances known as vApps (virtual applications) of the Eclipse RTP software, giving users access to a clinical training database for contouring and planning practice. All patients on the training database are anonymised training cases, obtained with patients’ consent. This configuration allowed up to 15 users to access the training database from Cape Town while being monitored in person in the classroom. An alternative solution had to be found to allow participants access from their *home departments* in such a way that their planning could be monitored and feedback could be given to participants in real-time.

**FIGURE 1:**
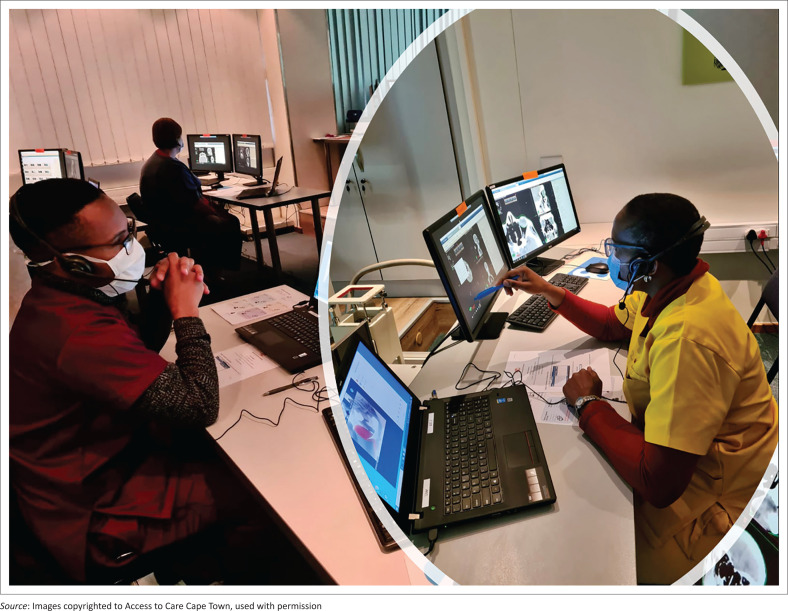
Classroom assistants monitoring remote teams during contouring exercises.

After investigation, the existing infrastructure was reconfigured to allow remote participants to access student vApp accounts through a web interface. Local faculty used VMWare Horizon View (https://www.vmware.com/) administration logins to duplicate the students’ screens and monitor all work in real-time. Communication with the students was facilitated through a separate laptop running Zoom. In this configuration, the lecturer could demonstrate a particular contouring or planning skill to the whole group by screensharing lecture material from the main planning computer screen. Zoom breakout rooms were then used to allow local classroom assistants to each interact directly with one to two teams as they attempted to master the new planning skill. Headsets were used to limit noise during the breakout sessions (Figure 1).

Participants require a stable internet connection for this solution to work. Lower bandwidth and speed, as well as high latency, may result in delays and interruptions in transmission. As a result, connectivity tests are essential for all participants to ensure full functionality during training.

#### Practical demonstrations

Previous courses included practical demonstrations of quality assurance procedures, patient setup protocols for different types of treatments (using phantoms), as well as general linear accelerator and controller computer operations. A solution to convey this information had to be found using the live-streaming platforms. As no network points were available inside the radiation bunkers, live-streaming was facilitated using a 100 m Category 6 network cable connecting an external university-based network point to a laptop inside the bunker. This allowed direct streaming of the different practical sessions to participants through Zoom. In addition to the streamed sessions, pre-recorded videos of practical demonstrations were used, to be followed by live discussion and question sessions.

#### Participant feedback

The free Jotform (https://www.jotform.com/) platform was utilised for all participant feedback questionnaires. As a result of the limited data processing functionality on the free platform, this was replaced with a paid subscription to utilise Survey Monkey as platform from the start of 2022 (https://www.surveymonkey.com).

Participant feedback showed that the level of teaching offered was appropriate, with some concerns about the time allocated for Eclipse work. This was primarily as a result of connectivity lag observed by some participants:

‘I struggled with a time-lag when using vCloud. I’m not sure whether it was my internet connection or the computer’s processing, but I found that whenever I clicked on something on Eclipse it took longer to respond than the host’s version.’ (Questionnaire response, September 2020)

The breakout room facility was found to be beneficial:

‘The break room sessions and the hands-on planning was so helpful it was a good bridge between the theory and putting things in place.’ (Questionnaire response, December 2020)

In general, participants felt better equipped to deal with challenges associated with the implementation of modern RT techniques:

‘I now have a broader view of the whole treatment planning process. More understanding of the basic principles of IMRT [*intensity modulated radiotherapy*] and VMAT [*volumetric modulated arc therapy*].’ (Questionnaire response, December 2020)

‘This course was very beneficial to my practice and has changed the way I work.’ (Questionnaire response, December 2020)

‘I think I will be much more adequately prepared to face the challenge of commissioning a new IMRT [*intensity modulated radiotherapy*] and VMAT [*volumetric modulated arc therapy*] capable linac, TPS [*treatment planning system*] and R&V [*Record and Verify system*] system.’ (Questionnaire response, December 2020)

However, the general consensus was that remote training should not replace in-person training:

‘The session was very helpful. However, it shouldn’t completely replace on-site trainings.’ (Questionnaire response, December 2021)

A summary of all computer-related modifications is given in Table 1.

**TABLE 1:** Computer-related modifications to allow for remote teaching during the COVID-19 pandemic.

Teaching tool	In-person training model	Remote training model	Cost implications	Importance
Teleconferencing facilities	No teleconferencing software used; single monitor used during lectures	Zoom platform, utilising breakout rooms. Web cameras added to all computers used by lecturers. Second monitor added to computers used by lecturers. Headsets procured for all facilitators.	Zoom (UCT): R1000.00 (€65.00) p.a. Computer hardware: R60 000.00 (€3800.00)	Essential Essential
Recording facilities	Classroom lectures recorded occasionally for internal use	Open Broadcaster Software (OBS) installed on all computers to pre-record material.	No additional cost	Essential
VERT^TM^ simulation system	Live demonstrations on VERT system during lectures, 2D or 3D	Demonstration sequences pre-recorded (2D) on OBS software and inserted in PowerPoint slides.	No additional cost	Advantageous
Strategic thinking whiteboard exercises	Physical whiteboards with magnetic printed items available for purchase (machines, staff, networking cables etc.) used to design a department. Excel spreadsheets for budget calculations. Presentation in person.	PowerPoint templates were pre-populated with images of machines, staff, network cables etc. available to purchase, allowing teams to design their departments. Excel spreadsheets were used for budget calculations. Presentation using Zoom.	No additional cost	Advantageous
Radiotherapy treatment planning computer access	The RTP server located in Switzerland, access to vApps via Dell Wyse clients located in the classroom. Access for 15 users. In-classroom monitoring and assistance during practical sessions.	The RTP server located in Switzerland, access for classroom assistants to vApps via Dell Wyse clients in the classroom, with duplicate student screens for continuous monitoring (admin logins). Additional laptop and Zoom were used for student communication. Student logins at home facilities using web interface to access the database in Switzerland. Access for a maximum of six remote teams per session.	Existing laptop software upgrades performed by UCT IT division, no additional cost. Reconfiguration of the vApp accounts carried out by Varian Education IT division, no additional cost. Reconfiguration of classroom computers carried out by UCT/CPUT staff, no additional cost.	Essential
Practical demonstrations	Demonstrations carried out in person in the radiotherapy department	Live streaming through a 100 m network cable for demonstrations, as well as pre-recorded material loaded on the Vula™ platform	Cat6 Ethernet cable: R2500.00 (€150.00)	Advantageous
Participant feedback	Pre- and post-course questionnaires were required from all participants, making use of the Jotform platform.	Continued use of the Jotform platform during 2020–2021, replaced with Survey Monkey in 2022 to obtain better statistics and graphics.	Survey Monkey: R5000.00 (€300.00) p.a.	Advantageous

RTP, radiotherapy treatment planning; UCT, University of Cape Town; CPUT, Cape Peninsula University of Technology; p.a., per annum.

### E-learning resource utilisation

#### LäraNära teaching platform

The LäraNära remote teaching platform allows participants to review core RT concepts before starting the A2C courses. No changes were required to the platform and all modules were still available to participants. However, this platform was not available for the uploading of local A2C training material. An alternative platform had to be found for this purpose.

#### The Vula™ learning management system

The University of Cape Town’s Vula™ platform was identified as a suitable platform for general course communication and hosting of A2C specific training material. The Vula™ is a Sakai learning management system (LMS) platform that allows for the creation of local project sites for each course (https://www.vula.uct.ac.za/portal). Participants are registered for the specific project site for the duration of the course as well as for three months after completion, allowing review of material before or after main lectures and sessions. No additional costs were charged by the University of Cape Town for the use of the Vula™ platform.

### Programming and curriculum changes

Previously, in-person training required participants to travel to Cape Town for the duration of the main training period. For the 3D CRT course, this resulted in 17 training days over a 21-day period. Cost associated with travel and accommodation for the duration of the course varied depending on country of origin, with the average cost about €9000.00 per team of three (RO, MP and radiation therapy technologist [RTT]). This included flights, accommodation and meals for the 3-week period, but excluded any course registration fees.

Participants and faculty often struggled with fatigue during the last week of the training block. The 3-week period also meant that visiting teams were away from their clinics for a protracted period, which could impact workflow. Similarly, it also resulted in pressure on the home department’s clinical workflows, as teaching faculty are employed on a full-time bases by the hospital and universities.

The move to a fully remote training platform allowed for these concerns to be addressed, by offering shorter training blocks extending over a longer period. As a result, the 17-day 3D CRT training course offered in 2021 was delivered between July and October including three 1-week blocks (one per month), each focusing on a specific clinical area. This principle was applied to all new courses developed during 2020–2021. The remote model also allowed for an overall reduction in cost associated with travel to the local facility, with only actual training/registration cost remaining.

Additional courses were also developed to address varying skills levels. With training no longer geographically localised, collaborations with international training partners were established with Planning for Africa (based in the United Arab Emirates) and RadiQa Developments (based in Italy). Five additional courses were introduced to A2C Cape Town between March 2020 and January 2022. The programme expanded beyond Africa to also include teams from Pakistan and the United Arab Emirates. Courses offered as of January 2022 include:

- 2D to 3D CRT course (3D CRT)
- Introduction to advanced techniques course (I2A)
- Advanced physics and planning course (PnP)
- Paediatric RT for LMICs (Paeds)
- Top to Toe – General RT technical planning skills (T2T)
- Top to Toe – Stereotactic RT planning skills (T2T:SRT)

Details of the in-person and remote courses offered between 2015 and January 2022 are given in Table 2.

**TABLE 2:** Overview of access to care Cape Town courses: January 2015–January 2022.

Course	Facilitator	Target audience	Programme scheduling	Teams
**3D CRT** *In-person* (2015–2020)	**UCT/CPUT** Cape Town	Departments transitioning from 2D to 3D CRT, limited knowledge of modern RT principles *Team: RO/MP/RTT*	LäraNära (8 modules) pre-course learning 17 days on-site, over 21-day period 3-month remote mentorship (email) to complete clinical protocols	Harare (Zim), Bulawayo (Zim), Kumasi (Gh), Accra (Gh), Yaoundé (Cam), Addis Ababa (2) (Eth), Misrata (Lib), Dar es Salaam (2) (Tan), Abuja (Nig), Lagos (Nig), Lusaka (Zam), Kampala (Ug)
**3D CRT** *Remote* 2021	**UCT/CPUT** Cape Town	Departments transitioning from 2D to 3D CRT, limited knowledge of modern RT principles *Team: RO/MP/RTT*	LäraNära (8 modules) pre-course learning Course spread over 4 months, 1 week per month Introduction day Week 1: Prostate (6 days) Week 2: Gynae (6 days) Week 3: Breast (6 days) 3-month remote mentorship (email/Zoom) to complete clinical protocols Course material hosted on Vula™	Lagos (Nig), Nairobi (2) (Ken), Eldoret (Ken)
**I2A** *Remote* 2020–2021	**UCT/CPUT** Cape Town	Departments preparing to implement modern radiotherapy techniques, including Field in field, IMRT and VMAT techniques *Team: RO/MP/RTT*	LäraNära (12 modules) pre-course learning Course spread over 4 months Introduction day I2A Block: 5 days PnP Block 1: 3 days PnP Block 2: 3 days Closing quiz day Course material hosted on Vula™	Harare (Zim), Dar es Salaam (Tan), Lusaka (Zam), Lagos (2 (Nig) Nairobi (Ken), Eldoret (Ken), South Africa (mixed team)
**PnP** *Remote* 2020–2021	**RadiQa Developments** Milan			
**Paeds** *Remote* 2021	**UCT/CPUT** Cape Town	Overview of paediatric radiotherapy principles for low- and middle-income countries *Team: RO/Planner*	3-day dry run (2021) Future course 5 days (2022)	21 participants from Africa invited to attend the dry run
**T2T** *Remote* 2021	**Planning for Africa** Abu Dhabi	Practical planning skills for 3D CRT, IMRT and VMAT *Team: MP/Planner*	2020: 10 weekend sessions over a 1-year period (Friday/Saturday morning) 2021: 8 weekend sessions over a 1-year period (Friday/Saturday morning)	Accra (Gh), Lagos (Nig), Eldoret (Ken), Lahore (Pak), Pietermaritzburg (SA), Nairobi (Ken)
**T2T:SRT** *Remote* New course 2022	**RadiQa Developments** Milan	Practical planning skills for hypo-fractionated stereotactic radiotherapy *Team: MP/Planner/RO*	2022: Introduction day 4 weekend sessions over a 1-year period (Friday/Saturday morning)	2022: Lagos (Nig), Nairobi (Ken), Cape Town (SA), Al Ain (UAE)

RO, radiation oncologist; MP, medical physicist; RTT, radiation therapy technologist; IMRT, intensity modulated radiotherapy; VMAT, volumetric modulated arc therapy; Zim, Zimbabwe; Gh, Ghana; Cam, Cameroon; Eth, Ethiopia; Lib, Libya; Tan, Tanzania; Nig, Nigeria; Zam, Zambia; Ug, Uganda; Ken, Kenya; SA, South Africa; Pak, Pakistan; UCT, University of Cape Town; CPUT, Cape Peninsula University of Technology.

### Key challenges of remote teaching

The following key concerns were raised by participants and local faculty:

#### Bandwidth and connectivity

Pre-course technical testing to confirm local computer setup, bandwidth and connectivity is essential to the success of the training. Teams require two computer screens, one to follow lectures on Zoom, and a second to work on the Eclipse system. Both screens must be of sufficient size and resolution (e.g. 1920 × 1080) to allow effective monitoring during exercises. Alternative locations (network access points) or methods of access should be discussed in advance, to be used in case of network failure. Where teams use different system configurations on different days, all must be tested.

#### Technical systems management

Technical assistance to teams during training, as well as management of the Zoom breakout rooms, should be performed by a dedicated person with appropriate information technology skills and the ability to troubleshoot. Guidelines for access should be given to participants prior to training and tested thoroughly before the start of the course.

#### Time zones

As training currently runs over a 7-h period per day for most courses, time zones of the participating teams and course facilitator should be considered and may be a limitation in terms of geographical reach of the programme. The alternative would be to run courses for fewer hours per day (3 h – 4 h per day), but extend the training to run over more months to still achieve the same amount of overall teaching time. This will allow more flexibility when teaching people over multiple time zones.

#### Online engagement

An important concern of teaching faculty is the ability to maintain a high level of engagement over the duration of the course. Course disruption because of teams logging in late or some participants failing to log in at all on certain days can be very disruptive to other teams and faculty.

### Alignment with the principles of modern medical education

The remote teaching model was finally reviewed against the five *Filetti principles of modern medical education*, as previously mentioned in the introduction. A summary of the analysis is given in Table 3.^14^

**TABLE 3:** Alignment with the principles of modern medical education.

Principle	A2C programme response
*Interactivity*	Interaction with participants is encouraged throughout training, utilising Zoom polling, verbal as well as chat function discussions, quiz sessions and team presentations. In addition to Zoom polling, Wooclap (https://www.wooclap.com/) is used to interact with students. Individual Zoom backgrounds reflecting the culture of each country encourages discussions between participants and faculty, making the online training feel more personal.
	Direct monitoring during contouring and planning sessions encourage discussion between teams and classroom assistants. As each assistant is only monitoring two teams, teams get individualised attention throughout the course.
*Bidirectionality*	Teams are given the opportunity to apply new planning or contouring skills in team assessment sessions. Every team is given the opportunity to present their case to assessors and to other attending teams, allowing for review of the new skills.
	Teams present their clinical protocols and departmental design projects to faculty and other teams, offering the opportunity for discussion and feedback.
*Blendedness*	Synchronous learning is performed through live streaming on the Zoom platform, while asynchronous learning is supported through the LäraNära and Vula™ platforms. The variation in training material, as well as the unique perspective offered through the simulation software, further supports blended learning.
*Transnationality*	The expansion of the programme to include academic collaborators from the UAE and Italy allows for a variation in teaching styles as well as clinical experience. The programme has expanded to include teams from Africa, the UAE and Pakistan.
*Up-to-dateness*	The Vula™ sites as well as all training material is reviewed on an annual basis to ensure it remains relevant.

*Source*: Filetti S, Grani G, Murat G, Saso L. Innovative medical eduation in the digital era. Filetti S, editor. Mc Graw Hill; 2022.

UAE, United Arab Emirates.

## Discussion

The virtualisation of the A2C programme allowed for continued teaching during the pandemic, with 18 teams attending remote teaching courses between April 2020 and January 2022 as well as completion of the outstanding modules for the team from Uganda who had to leave the last in-person course prematurely in March 2020.

Virtualisation of the A2C programme has provided many opportunities and insights into RT practical training and on-line educational teaching models generally.

Computer hardware and software changes are summarised in Table 1. Although careful planning was required, the number of modifications was achieved relatively easily and at a manageable cost, and the main concerns related to upskilling the home team in complicated information technology (IT) and technical troubleshooting.

An IT coordinator, although always necessary for A2C courses, has become an increasingly critical part of all the courses, to ensure that courses proceed smoothly. Pre-recording of lectures is an effective way of ensuring that absent lecturers (whether because of COVID-19 or for other reasons) can continue to deliver a high-quality product in a consistent way. The use of the VERT™ virtual simulation system was limited due to the fact that it is still on the Windows 7 operating system, which is considered a network security risk. A work around was found by using pre-recorded video material, which worked well.

The strategic thinking departmental design team exercise is one area where the on-line model may not work as well as the face-to-face version. This exercise was initially run as a competitive game, with teams learning from other team members and benefiting from the facilitator’s input. The on-line version of the exercise does not have the same impact, and going forward, consideration will be given to making this a separate stand-alone course, with more time available. It is considered a critical part of A2C.

The RT treatment planning computer access strategy has proven to be successful. However, a key concern for online training raised by both teaching faculty and course attendees was found to be stable internet connectivity. Connectivity issues during training can be very disruptive and needs to be addressed in the background while training continues. For this reason, it is essential to have one person dedicated to always managing the online platform, as this cannot be performed by the instructors while they are lecturing. Alternative methods of access must be tested in advance, to prevent one or multiple teams losing connectivity during training. Reserve training days should also be considered where all connectivity is lost because of international internet service provider failures.

Interestingly, the practical demonstrations from the live RT machine bunker were one of the biggest concerns regarding the virtual courses. However, the new combination of live streaming and pre-recorded videos works very well. The annual upgrade of all videos is necessary to ensure up to date material.

Increasing awareness of resources available, as well as familiarity with some of the on-line platforms has become a positive off shoot of the courses, accelerating adoption of new platforms. New skills are taken into the courses and into the home department.

One example of this is the increased use of the Vula™ platform. Although available for free for several years prior to the pandemic, it is only now routinely incorporated into training, making the courses more modern, and allowing access to the training material both before and after the modules were taught, according to preference. The active assessment of not only the training material but also the training methodology, also allowed for a much better alignment with the principles of modern medical education as detailed in Table 3.

The programming and curriculum changes are generally considered to have been an improvement on 17 continuous days of learning. The advantages to both the home teaching team and the visiting team from an LMIC relate to less workflow issues because of missing staff for long periods. However, there is a concern relating to team engagement when courses run over multiple weeks of training. As teams are still located in their home departments, there is a considerable risk of clinical pressures pulling them away from the training. This is particularly of concern for the RO in the team, and the concern needs to be addressed with each department’s managers in advance, to ensure maximum attendance of the courses by the full team.

## Conclusion

Although the pandemic has brought many challenges to cancer care and cancer education, it has also opened new avenues to teaching and training. The immediate decision to virtualise the A2C training platform has allowed the programme to continue to function optimally and to reach further than ever before despite the COVID-19 pandemic. Keeping participants engaged on the online platform for extended periods of time remains a big challenge, especially when participants are also required to continue fulfilling clinical duties while courses are run. This was evident from the number of ROs who excused themselves from training sessions because of clinical obligation. Hospital management’s support during training is critical if participants are to make the most of the training opportunities. Despite these challenges, there are considerable advantages in terms of geographical reach as well as financial and time savings in terms of travelling for in-person attendance of courses. It is therefore very unlikely that the A2C training programme will revert to face-to-face teaching for all courses in the future. However, there are certain courses, such as the departmental design and procurement training, that will gain from in-person training. For this reason, some courses may in future be delivered in a hybrid format where a part of the training is performed remotely, with only a short training block performed in person.

## References

[1] Worldometer. Worldometer [homepage on the Internet]. 2022 [cited 2022 Feb 09]. Available from: https://www.worldometers.info/coronavirus/

[2] International Agency for Research on Cancer. GLOBOCAN 2020 [homepage on the Internet]. [cited 2022 Feb 09]. Available from: https://gco.iarc.fr/today/data/factsheets/populations/900-world-fact-sheets.pdf

[3] World Health Organization. World Health Organization Europe [homepage on the Internet]. 2022 [cited 2022 Feb 09]. Available from: https://www.euro.who.int/en/media-centre/sections/statements/2022/statement-cancer-services-disrupted-by-up-to-50-in-all-countries-reporting-a-deadly-impact-of-covid-19

[4] Martei YM, Rick J, Fadelu T, Ezzi MS. Impact of COVID-19 on cancer care delivery in Africa: A cross-sectional survey of oncology providers in Africa. JCO Glob Oncol. 2021;7:368–377. 33689484 10.1200/GO.20.00569 PMC8081536

[5] International Atomic Energy Agency. DIRAC [homepage on the Internet]. [cited 2022 Feb 09]. Available from: https://dirac.iaea.org/Query/Map2?mapId=0

[6] Atun R, Jaffrey A, Barton B, Bray F. Expanding global access to radiotherapy. Lancet Oncol. 2015;16(10):1153–1186. 26419354 10.1016/S1470-2045(15)00222-3

[7] Elmore SNC, Polo A, Bourque JM, Pynda Y, Van der Merwe D, Grover S. Radiotherapy resources in Africa: An international IAEA update and analysis of projected needs. Lancet Oncol. 2021;22(9): E391–E399. 34478675 10.1016/S1470-2045(21)00351-X PMC8675892

[8] Rodin D, Longo J, Sherertz T, Shah MM, Balagun O, Wending N. Mobilising expertise and resources to close the radiotherapy gap in. Clin Oncol. 2016;29(2):P135–P140. 10.1016/j.clon.2016.11.008 27955997

[9] Burger, H., Wyrley-Birch, B., Joubert, N. et al. Bridging the Radiotherapy Education Gap in Africa: Lessons learnt from the Cape Town Access to Care Training Programme over the past 5 years (2015–2019). J Canc Educ. 2022;37:1662–1668. 10.1007/s13187-021-02010-5 PMC8084586 33928527

[10] International Atomic Energy Agency. Transition from 2-D radiotherapy to 3-D conformal and intensity modulated radiotherapy. Vienna: International Atomic Energy Agency (IAEA) 2008.

[11] Degerfalt J, Sjostedt S, Fransson P, Kjellen E, Werner M. E-learning programs in oncology: A nationwide experience from 2005 to 2014. BMC Res Notes. 2017;10:39. 28086959 10.1186/s13104-017-2372-8 PMC5234116

[12] Van Dyk J. The modern technology of radiation oncology, volume 3: A compendium for medical physicists and radiation on oncologists. Madison, WI: Medical Physics Publishing, 2013; p. 361–412.

[13] Tanda L. Coronavirus in SA: Ramaphosa declares national ‘state of disaster’, imposes travel bans. News24 [serial online]; 2022 [cited 2022 Nov 28]. Available from: Coronavirus in SA: Ramaphosa declares national ‘state of disaster’, imposes travel bans

[14] Filetti S, Grani G, Murat G, Saso L. Innovative medical education in the digital era. Filetti S, editor. New York, NY: Mc Graw Hill; 2022.

[15] Grosser J, Bientzle M, Shiozawa T, Hirt B, Kimmerle J. Acquiring clinical knowledge from an online video platform: A randomized controlled experiment on the relevance of integrating anatomical information and clinical practice. Anat Sci Educ. 2018;12(5):478–484. 30347523 10.1002/ase.1841

[16] Grosser J, Bientzle M, Shiozawa T, Hirt B, Kimmerle J. Observing interprofessional collaboration: Impact on attitude and knowledge acquisition. Anat Sci Educ. 2021;14(4):452–459. 32735751 10.1002/ase.2007

